# Physical therapy options for knee osteoarthritis: A review

**DOI:** 10.1097/MD.0000000000038415

**Published:** 2024-07-26

**Authors:** Xinxin Ni, Lianxin Hu, Xun Zhang, Zefeng Wang, Cong Yan, Laurent Peyrodie, Minghai Lin, Xinyue Wu, Huajun Wang, Shijia Hu

**Affiliations:** a Huzhou University, Huzhou, Zhejiang, China; b Institut Supérieur d’Électronique de Paris, Paris, France; c Beijing University of Chinese Medicine, Beijing, China; d ICL, Junia, Université Catholique de Lille, LITL, F-59000 Lille, France.

**Keywords:** knee osteoarthritis, moxibustion therapy, physical therapy, recovery

## Abstract

In recent years, osteoarthritis of the knee, a common degenerative joint disease, often occurs in the elderly population. This disease has a significant impact on the quality of life of patients. For treating knee osteoarthritis, physical therapy is highly regarded as a very effective treatment method. This article delves deeply into commonly used physical therapy methods and analyzes their therapeutic effects, cost-effectiveness, and applicability, aiming to find treatments with broader applicability and better cost-effectiveness. The goal is to help a large number of patients effectively alleviate the discomfort caused by knee osteoarthritis, enhance the clinical therapeutic effects, and introduce home treatment methods to reduce financial burdens. The article also compares various physical therapy methods and finds that moxibustion and electrotherapy are more suitable for home use. Other treatment methods provide a reliable scientific basis for patient treatment.

## 1. Introduction

This text focuses on the application of various physical therapy methods in the treatment of knee osteoarthritis (KOA), exploring their effects in alleviating pain, improving function, and promoting recovery.

Current research has proven that conditions such as KOA, periarthritis of shoulder, and cervical spondylosis can be managed through extracorporeal physical therapy.^[1–3]^ Among them, KOA is one of the most common chronic diseases worldwide, and the number of patients affected by KOA is increasing both domestically and internationally, imposing a significant burden on individuals and society.^[4]^

KOA is a common chronic joint disease, with the main symptoms being pain, stiffness, and swelling. Pain often intensifies after walking or staying in the same position for a long time, especially at night or when getting up in the morning. Joint stiffness makes it difficult to bend or straighten the knee, affecting mobility. Synovitis causes swelling and pain around the joint, and this swelling can enlarge the knee, affecting daily activities.

According to epidemiological surveys at home and abroad, 19% of middle-aged and elderly people in the U.S. suffer from KOA.^[5]^ As age increases, the prevalence of KOA also rises.^[6–8]^ In China, the prevalence of KOA is 18%,^[9]^ with a rate of 16.5% for those aged 40 to 49, 30.2% for those aged 50 to 59, 37.9% for those aged 60 to 69, and as high as 47.5% for those over 70-years old.^[10]^ This disease severely affects the quality of life of patients and also places a significant burden on families and society.

Although there are numerous studies on physical therapy for KOA, there is still a need for more comprehensive comparative research on the effects of different physical therapies in treating KOA. Based on this, this paper builds upon previous research to compare and analyze the results of various physical therapy methods, including shortwave therapy, therapeutic ultrasound, and acupuncture among others. These physical therapy techniques can effectively improve disease symptoms and accelerate the recovery process, and compared to drug treatment, physical therapies have fewer side effects, making them a safer treatment option.

## 2. Physical therapy approaches for knee osteoarthritis patients

In recent years, physical therapy has been unanimously recommended by osteoarthritis guidelines both domestically and internationally due to its therapeutic characteristics and reliability as one of the primary treatment modalities for KOA. Latest research has also found that physical therapy can enhance the physiological condition and functional recovery of KOA patients, improving their quality of life.^[11]^ Currently, physical therapy is also extensively applied in clinical medical institutions and physiotherapy clinics. Some physical therapy techniques have even become popular in households, becoming a routine part of treatment.^[12]^ According to various osteoarthritis treatment guidelines and research directions both domestically and abroad, physical therapies for KOA include acupuncture, moxibustion, therapeutic ultrasound, shortwave therapy, pulsed electromagnetic field (PEMF) therapy, low-intensity laser therapy, low-frequency electrotherapy, medium-frequency electrotherapy, and high-frequency electrotherapy.

### 2.1. Acupuncture therapy

Acupuncture is a traditional Chinese medical therapy, which involves the insertion of fine needles into specific acupoints on the patient’s body. Through lifting, thrusting, and twisting techniques, local tissues are stimulated to regulate the body’s Yin and Yang and balance the flow of Qi and blood in the meridians. This therapy has been widely applied in China for thousands of years. Currently, acupuncture has been extensively used in the treatment of osteoarthritis and has become an integral part of its therapeutic approach. The significant role and applicability of acupuncture in treating KOA have reached consensus among experts.^[13]^ In a systematic analysis by Ben-Arie et al^[14]^ a substantial amount of research data demonstrated the safety and efficacy of acupuncture in treatment, capable of effectively alleviating pain in the short and medium term. Table 1 provides the feasibility analysis data for acupuncture treatment of KOA. However, the effects of acupuncture on relieving pain in the medium to long term are still under debate, and more large-scale, high-quality experimental data is needed for research and comparison to yield accurate results.

**Table 1:** Analysis of acupuncture treatment for KOA.

Test subjects	Research design	Results
100 professionals and nonprofessionals^[13]^	Semi-structured interview	Treatment has an efficacy rate as high as 92%
Search the 4 major types of databases^[14]^	Meta-analysis	Effective in the short and medium term
Establishing an animal model for KOA^[15]^	Acupuncture combined with drug therapy experiment	Treatment efficacy rate found to be as high as 90%
62 patients with KOA^[16]^	Randomly divided into 2 comparison groups	Treatment efficacy rate as high as 90.3%
56 patients with KOA^[17]^	Randomly divided into 2 comparison groups	Pain effectively relieved with good tolerance

KOA = knee osteoarthritis.

### 2.2. Moxibustion therapy

In addition to acupuncture, there is another similar therapy – moxibustion. Moxibustion is an ancient Chinese medical treatment. It stimulates specific parts of the body’s surface through the heat produced by burning mugwort leaves, serving as a way to regulate the body’s “Qi” (vital energy) and promote the function of internal organs. Compared to traditional acupuncture, moxibustion is gentler, painless, and is increasingly accepted and loved by many. Meanwhile, research has also found that moxibustion has a certain effect in thermotherapy for KOA. Table 2 presents an analysis of moxibustion’s effect on KOA, though a comprehensive system has yet to be established.

**Table 2:** Analysis of moxibustion treatment for KOA.

Test subjects	Research design	Results
40 New Zealand big-eared White rabbits^[18]^	Utilized 4-group comparison experiment method	Inhibited the aggregation of knee joint cartilage inflammation
Search databases^[19]^	Meta-analysis	Efficacy rate as high as 95%
50 patients with KOA^[20]^	Control experiment	Efficacy rate as high as 100%
48 patients with KOA^[21]^	Control experiment combined with Gua Sha	Efficacy rate as high as 97.9%

KOA = knee osteoarthritis.

In an experiment conducted by Yuan et al,^[22]^ they conducted a comparative study between moxibustion and therapeutic western medicines. The results showed that the therapeutic effects of moxibustion were better and safer than those of western medicine. Although this experiment has limitations and more large-scale, high-quality research is needed to obtain further results, it does to some degree prove that the moxibustion treatment for KOA is effective and safe. Moxibustion does not have as significant an effect as traditional acupuncture in KOA treatment, however, the combined treatment of moxibustion and other drugs can achieve significant differences before and after treatment. For example, Dai et al’s^[23]^ comparative experiment between the moxibustion method and the daily oral intake of 0.2-gram Celecoxib capsules (Approval number: National Medicine Permission J20120063, Manufacturer: Pfizer Pharmaceuticals LLC/Pfizer Pharma Limited) found that the combination of moxibustion and drugs was more effective in relieving pain than drugs alone.

Taking this into consideration, we can deduce that moxibustion does have a positive effect during its treatment, but the exact extent of this effect still needs further verification. Currently, moxibustion can be considered as an intervention method for KOA treatment.

### 2.3. Therapeutic ultrasound

Ultrasonic waves are mechanical vibration waves with a frequency exceeding 20 kHz, which have cavitation, thermal effects, and mechanical effects.^[24]^ The application range of ultrasonic waves is very broad, not only in medicine but also in industries such as food processing. The main applications of ultrasonic waves in medicine are in imaging and treatment. They can be used to break down tumors and stones and clear thrombi from blood vessels. Ultrasonic waves are one of the physical therapies for treating KOA. Its imaging function is the preferred method of examination for KOA, primarily used for assessment and monitoring of osteoarthritis. Furthermore, using therapeutic ultrasound for KOA can also reduce the tension in muscles and connective tissues, thereby alleviating KOA pain and aiding in the recovery of knee joint dysfunction. Table 3 lists the analysis of this treatment method.

**Table 3:** Analysis of ultrasonic therapy treatment for KOA.

Test subjects	Research design	Results
120 patients with KOA^[25]^	Divided into 3 comparison groups	Reduced pain and promoted cartilage repair in the joint
70 patients with KOA^[26]^	Control experiment combined with acupuncture	Total efficacy rate as high as 94.29%
30 patients with KOA^[27]^	Control experiment combined with warm needle therapy	More pronounced therapeutic effect

KOA = knee osteoarthritis.

Further research indicates that continuous therapeutic ultrasound can effectively relieve pain in KOA patients and enhance their mobility.^[28–30]^ Ultrasonic waves are not only used for KOA treatment. Many cases involve the use of ultrasonic waves in treatments; currently, it’s mainly used to address other inflammatory-related issues. However, the number of studies available is still limited, and some experiments have found that the clinical efficacy of ultrasonic waves in treating KOA is of low quality.^[31]^

From this, we can deduce that ultrasonic treatment for KOA is effective, but may have limitations. The specific effectiveness needs substantial experimental data for support to provide a comprehensive analysis report. Meanwhile, based on known data, we can infer that the therapeutic effect of ultrasonic waves is generally greater than that of moxibustion, but its comparison with acupuncture still awaits further data.

### 2.4. Shortwave therapy

Shortwave therapy is a treatment method that uses the high-frequency vibrations of electromagnetic waves to produce thermal effects and is widely applied in the medical field. This therapy can stimulate the patient’s blood circulation, promote tissue repair, and can also relieve pain and eliminate inflammation. In short, shortwave therapy is a type of physical therapy similar to magnetic therapy. According to relevant data, shortwave therapy also has significant effects in treating arthritis. In fact, shortwave therapy stimulates blood circulation in the affected area, promoting tissue repair and regeneration, thus relieving pain and inflammation. The therapeutic effects are shown in Table 4.

**Table 4:** Analysis of shortwave therapy treatment for KOA.

Test subjects	Research design	Results
23 patients with KOA^[32]^	Direct observation method	Overall efficacy rate of 86.96%
80 patients with KOA^[33]^	Controlled experiment	Treatment group efficacy rate of 75% is higher than the control group
60 patients with KOA^[34]^	Treatment combined with acupuncture	Combined group effect > Shortwave group
76 patients with KOA^[35]^	Controlled experiment	Overall efficacy rate of 86.8%

KOA = knee osteoarthritis.

Additionally, shortwave therapy can increase the elasticity of soft tissues, making joints more flexible, thereby improving motor function. For treating KOA, the efficacy of shortwave therapy is evident. When treating KOA, electromagnetic radiation of 27.12 MHz is generally used, applying continuous or pulsed shortwave diathermy to produce either “thermal” or “nonthermal” effects.^[36]^ According to a survey of 116 senior physiotherapists in the physiotherapy departments of 41 Irish hospitals, it was found that both pulsed sound wave therapy (PSWD) and continuous sound wave therapy have their respective advantages when treating KOA.^[37]^

Specifically, continuous sound wave therapy has shown good therapeutic effects in treating chronic KOA, while PSWD is considered by most physiotherapists as an effective method for treating acute KOA. Moreover, based on current data, PSWD is generally seen by therapists as the preferred method for treating KOA. Although shortwave therapy can effectively treat KOA for most patients, some believe that shortwave therapy might aggravate the condition. Considering safety and economics, the technology of shortwave therapy is relatively advanced, with a high treatment threshold, and its technology and equipment level are constantly updated and have not yet matured fully. Therefore, some people might not be suitable for shortwave therapy and might even worsen their condition. Given the above considerations, compared to acupuncture physiotherapy, moxibustion, and ultrasonic physiotherapy, shortwave therapy is not suitable as the primary treatment method for KOA.

### 2.5. Pulsed electromagnetic field therapy

PEMF therapy is based on the principles of bioelectromagnetism, using the properties of pulsed magnetic fields and pulsed electric fields to stimulate human tissues. The pulsed magnetic field induces weak currents by causing changes in the electric field. These weak currents can regulate the electrophysiological processes inside and outside the cells, promoting cell metabolism, improving blood circulation, and providing anti-inflammatory effects. This treatment method is widely used for the repair and bone reconstruction in KOA, using the above mechanism to relieve pain, reduce inflammation, and restore knee joint function. Bao et al^[38]^ found through research that the biological effects of magnetic fields are closely related to their frequency parameters and that there is an optimal magnetic field parameter “window.” One to hundred Hz is the frequency parameter range of PEMF currently used in clinical and basic research, and it has received good biological effect feedback. Fan^[39]^ also conducted relevant research on the treatment of bone diseases with PEMF. The study found that after the action of PEMF, the increase in bone density in the bone stem area with lower bone formation activity was more noticeable.

This provides important clues to the mechanism of PEMF in the treatment of KOA. However, although the therapeutic effect of PEMF on KOA has been confirmed, the mechanism of action of PEMF on bone cells is not yet clear from the bone cell level. The existing literature and research materials do not have consistent results, and it can only be inferred from the treatment results that PEMF have a regenerative and restorative effect on the bone. Using PEMF for the treatment of KOA is undoubtedly an innovative integration. It is also a new treatment method. Given the serious aging population in our country and the rising incidence of KOA, it is believed that this treatment method will have a broad application prospect.

### 2.6. Low-intensity laser therapy

Laser therapy can be divided into low-intensity laser therapy and high-intensity laser therapy. Although high-intensity laser therapy has stronger penetration and higher energy intensity compared to low-intensity laser therapy, it cannot be denied that high-intensity laser therapy might have a better therapeutic effect on KOA than low-intensity laser therapy. However, since high-intensity laser therapy has only recently been introduced to clinical practice and lacks a vast amount of data to support it, this section mainly discusses low-intensity laser therapy.

Low-intensity laser therapy is a commonly used physical therapy method and is widely applied in the management and rehabilitation of KOA. This therapy is based on the biostimulatory effects of lasers, using laser beams of specific wavelengths to irradiate the knee joint area. These laser beams penetrate the skin at low energy levels, get absorbed by tissues, and are transformed into biochemical energy. This biochemical energy can stimulate cellular metabolic processes, improve blood circulation and anti-inflammatory effects, thus promoting tissue repair and rehabilitation, reducing inflammation, and alleviating pain. Table 5 describes the analysis of treating KOA using low-intensity laser therapy. Like shortwave therapy, low-intensity laser therapy has the drawback of requiring professional medical personnel for operation, which makes the cost and barriers to the therapy relatively higher.

**Table 5:** Analysis of low-intensity laser therapy treatment for KOA.

Test subjects	Research design	Results
Searched database^[40]^	Meta-analysis	Effectively reduces pain and enhances knee joint function
89 patients with KOA^[41]^	Controlled experiment	Effectively reduced patient healing time
60 patients with unilateral KOA^[42]^	Combined acupuncture controlled experiment	Combined group > Laser group

KOA = knee osteoarthritis.

When treating KOA with laser therapy, the usual treatment frequency is 2 to 3 times a week, lasting 10 to 15 minutes each time, for a course of 4 to 6 weeks. Chen C and others^[40]^ used a meta-analysis on the therapeutic effects of both low-intensity and high-intensity lasers combined with rehabilitative exercises on KOA. The analysis found that both types of laser therapies could have a positive effect on KOA treatment. Current data cannot compare the therapeutic effects of the 2. However, theoretically speaking, this article retains the aforementioned view that high-intensity might have a better therapeutic effect on KOA than low-intensity laser therapy. To study the therapeutic effects of low-intensity lasers on KOA, Alfredo et al^[43]^ analyzed experiments conducted on 43 KOA patients. The results found that long-term low-intensity laser therapy can achieve pain reduction, lower disability rates, and reduce medication intake within 6 months.

### 2.7. Low-frequency electrotherapy

Low-frequency electrotherapy is based on the principles of electrophysiology, stimulating tissues through low-frequency currents. Such stimulation can alter the electrical activity of the cell membrane, affecting neural transmission and cellular metabolism, thereby producing pain relief and physiological effects. Low-frequency electrotherapy often uses currents with frequencies ranging from 1 to 100 Hz for treatment. While there are various treatment methods, transcutaneous nerve stimulation is commonly used. Compared to other physical therapy methods like ultrasound, lasers, short-waves, and pulsed therapies, the threshold for low-frequency electrotherapy is relatively low. It does not require extensive professional skills and advanced equipment; pinpointing the acupoints accurately is essential. Furthermore, the patch design used in low-frequency electrotherapy minimizes skin damage, eliminating the need for punctures. The patches are local, needing only to be placed near the acupoints to achieve effects similar to acupoint acupuncture.

Xiang et al^[44]^ conducted an analysis of randomized controlled trials on transcutaneous nerve stimulation for KOA pain relief from the Cochrane, Pub Med, and Embase databases. The analysis found no significant difference between acupoint stimulation by low-frequency electrotherapy and the control group. In the experimental results, 66.7% of patients in each group achieved pain relief. Due to the subjectivity of the experimenters, this isn’t sufficient to prove the pain-relieving effects of low-frequency electrotherapy. Xia^[45]^ randomly divided 70 patients with KOA who had no significant difference in inflammatory factor levels into 2 groups. One group used drug therapy as the control group, while the other group added low-frequency electrotherapy on the basis of drug therapy. After treatment, both groups improved. Compared with the first group, the level of inflammatory factors in the second group was significantly reduced. It can be understood that drug therapy has certain clinical efficacy, and it is more recommended to be supplemented with low-frequency physiotherapy. Chunyan et al^[46]^ and others pointed out in the design of mid and low-frequency electrotherapy and pain assessment systems that low-frequency electrotherapy can be used for superficial pain treatments. Most electrotherapy devices on the market use low-frequency electrotherapy as the primary treatment method. There are also devices that use mid-frequency electrotherapy, and some use a mix of both. In terms of quantity, low-frequency electrotherapy devices dominate the market. However, the treatment effects of low-frequency electrotherapy as described in various literatures are controversial. Currently, there isn’t enough data to support any viewpoint, so more data is needed in the future to prove its therapeutic effect.

### 2.8. Mid-frequency electrotherapy

The frequency range of mid-frequency electrotherapy is wide, ranging between 1 and 100 kHz. Mid-frequency therapy has various therapeutic effects and application fields for KOA. By altering the current’s frequency, waveform, intensity, and duty cycle, it stimulates nerves, muscles, and tissues, thereby promoting blood circulation, relieving pain, and facilitating tissue repair. Compared to low-frequency electrotherapy, mid-frequency electrotherapy has a definitive analgesic effect, and its therapeutic effect is significantly better than that of low-frequency electrotherapy.^[47]^

Xiaojun J et al^[48]^ proposed 3 traditional Chinese medical treatments, which includes medium-frequency electrotherapy. In their experiment, they divided 90 patients (134 knees) with KOA into 3 groups. The first group took oral Chinese medicine, and the second group added medium-frequency electrotherapy based on the first group. After clinical comparison, it was found that the addition of medium-frequency electrotherapy was more effective than drug treatment alone. This shows that medium-frequency electrotherapy is to some extent beneficial in relieving pain in KOA patients. Ming et al^[49]^ included 76 KOA patients and randomly divided them into a control group and an observation group. The experimental results found that the overall clinical effectiveness of low-medium frequency electrotherapy reached 89.47%, which is higher than the 68.42% of the drug group. The difference is statistically meaningful (*P* < .05). Therefore, it can be concluded that low-medium frequency electrotherapy can indeed better relieve pain and promote the recovery of knee joint function. This article also disassembled and analyzed some equipment and made some related instruments. When low-frequency electrotherapy is applied with a frequency below 10 Hz and a small current, it is similar to the “Electric Chewing Gum” device in the early 20th century, which can serve as a prank toy. If a slightly larger current is used, it can produce the “percussion” effect claimed by most of the electrical therapy instruments on the market. When the frequency exceeds 100 Hz, regardless of whether a large or small current is used, fingers start to lose autonomous control and produce a numbing effect. If mid-frequency electrotherapy uses a small current, it produces a slight numbing effect; if a slightly larger current is used, it feels like gua sha scraping. The above is a brief description and effect summary of these self-developed devices. However, comparative studies on mid-frequency and low-frequency electrotherapy are still limited, and further research is needed to clarify their relative advantages and applicability in KOA treatment.

### 2.9. High-frequency electrotherapy

High-frequency electrotherapy operates within a frequency range of 100 kHz to 10 MHz. By employing high-frequency currents, this therapy aids in pain management, muscle relaxation, and tissue repair. High-frequency electrotherapy is widely used in fields such as rehabilitation, sports injury recovery, and pain management. During the treatment process, specialized electrotherapy instruments and electrode patches transmit the high-frequency currents to the affected area or treatment zone. By adjusting the frequency and waveform of the current, high-frequency electrotherapy induces stimulation, which then enhances blood circulation, alleviates pain, promotes tissue repair, and relaxes muscles. Compared to other electrotherapy methods, high-frequency electrotherapy possesses unique characteristics: the sensations from its stimulation are relatively intense, and patients might feel significant stimulation or vibrations. Also, due to its intensity, the treatment duration for high-frequency electrotherapy is typically shorter, lasting several to around 15 minutes each session.

However, high-frequency electrotherapy also has some disadvantages. Due to its intensity and safety, patients should follow the guidance of doctors and operate it correctly when using it. At the same time, compared with low-frequency electrotherapy, experiments of high-frequency electrotherapy can significantly relieve the pain of KOA patients.^[50]^ This is because high-frequency electrotherapy can produce thermal and nonthermal effects in deep tissues, promote the absorption of inflammatory exudates, improve the nutrition supply of joint cartilage, reduce the tension around the joints and ligaments, adjust the secretion of cytokines, thus achieving the purpose of analgesia, anti-inflammation, and slowing down cartilage degeneration.^[51]^ Yanmei and Lining^[52]^ randomly divided 85 KOA patients into 3 groups, namely high-frequency electrotherapy group, exercise group, and high-frequency electrotherapy plus exercise group. The experimental results found that high-frequency electrotherapy can relax soft tissue, and the treatment effect is better than the exercise group. Of course, the effect of combined treatment is better than that of the high-frequency electrotherapy group. Compared with other physical therapy methods such as ultrasound, laser, shortwave, and pulse therapy, high-frequency electrotherapy has more promotional value due to its potential clinical and economic benefits. Table 6 describes the analysis of the effects of treating KOA using electrotherapy at 3 different frequency bands.

**Table 6:** Analysis of electrotherapy treatment for KOA.

Electrotherapy frequency	Research design	Test subjects	Results
Medium-frequency	Controlled experiment	127 patients with KOA^[53]^	Significant therapeutic effect
Low-medium frequency	Controlled experiment	76 patients with KOA^[54]^	Promotes joint function recovery
High-frequency	Systematic review and meta-analysis	Searched database^[55]^	Noticeable improvement

KOA = knee osteoarthritis.

## 3. Comparative analysis of knee osteoarthritis physical therapy protocols

### 3.1. Comparison of acupuncture, therapeutic ultrasound, shortwave therapy, and pulsed electromagnetic field therapy

Acupuncture and therapeutic ultrasound are 2 methods used for KOA treatment. Acupuncture, by stimulating specific acupoints, regulates the balance of Yin and Yang in the human body, providing a safe and effective short and medium-term pain relief effect. However, its long-term pain relief efficacy requires further study. In contrast, moxibustion, as a milder therapy, is not as effective as acupuncture in treating KOA, but it produces noticeable differences when combined with medications. Therapeutic ultrasound can be used for KOA imaging evaluation and pain relief, but current research on it is limited, and its clinical efficacy has certain constraints. Overall, both acupuncture and therapeutic ultrasound show some effectiveness in KOA treatment, but a thorough evaluation of their effects and a comparative assessment still require further in-depth research and substantial experimental data support.

Compared to acupuncture, shortwave therapy has shown significant therapeutic effects in treating KOA, but its technology and equipment levels still need further development and are not suitable as a primary treatment option. When choosing a treatment method, one should determine the most appropriate therapy based on individual circumstances and doctors’ recommendations. The combined treatment of acupuncture and shortwave therapy can also produce good therapeutic effects on KOA. Erhong et al^[56]^ took 60 KOA patients from Guangdong 39 Neurology Hospital as research subjects. After using warm needle acupuncture combined with shortwave treatment, they found that the overall treatment effectiveness rate reached 96.66%. Yanling et al^[57]^ pointed out in their article that when acupuncture is combined with shortwave therapy to treat KOA, the combined treatment effectively reduces the levels of tumor necrosis factor-α in the serum of KOA patients. From this, we can infer that each physical therapy method has its advantages. Properly combining different physical therapies with different mechanisms can achieve even better therapeutic effects.

PEMF therapy and shortwave therapy are 2 common physical therapy methods used to alleviate KOA symptoms. They have some similarities but also differ in various aspects. Both PEMF therapy and shortwave therapy can serve as adjunctive treatments for KOA. PEMF therapy promotes bone cell metabolism and repair by modulating the electrophysiological processes within and outside cells, while shortwave therapy promotes blood circulation and tissue repair by producing either a thermal or nonthermal effect. However, the threshold for both treatments is relatively high, and the choice of therapy should be based on individual circumstances and doctors’ recommendations.

### 3.2. Combined treatment of low-intensity laser, acupuncture, and electrotherapy

Low-intensity laser and acupuncture also have related research literature on combined therapy. However, the combination of low-intensity laser and acupuncture differs from acupuncture combined with shortwave therapy. If the latter is said to be complementary, the former is integrative. AL RASHOUD AS and others^[58]^ applied a laser with an energy density of 4 J/cm^2^ to the knee joint acupoints of 26 KOA patients. After 3 weeks of treatment, they found a significantly positive therapeutic effect compared to the control group. Lin et al^[59]^ discussed clinical research on low-intensity laser acupuncture treatment for KOA from 15 to 20 years in their review. They found that low-intensity laser acupuncture has a significant therapeutic effect on pain and function in treating KOA. This is a more innovative form of integration, cleverly combining the advantages of both, and these research results have proven its feasibility.

Similar to the ingenious combination of low-intensity lasers and acupuncture, there’s also the combination of electrotherapy and acupuncture. However, it’s not the low, medium, or high-frequency electrotherapy mentioned earlier. Instead, it stimulates acupuncture points by simulating acupuncture techniques, achieving a “noninvasive” acupuncture treatment. The electrotherapy combined with acupuncture described here involves adding a small electrical current on the basis of acupuncture. In this method, the mechanism used involves different waveform pulse currents to replace manual needle twirling, achieving the stimulation effect. Plaster et al^[60]^ conducted a controlled experiment on KOA patients involving 60 subjects. The results showed that both the acupuncture group and the electroacupuncture group had significant therapeutic effects, but the acupuncture group was slightly better in relieving pain and improving knee function than the electroacupuncture group. However, considering the variability in acupuncture techniques from one therapist to another, this study couldn’t make an accurate comparison between acupuncture and electroacupuncture. It can be said that this combined treatment method offers a new option for KOA.

### 3.3. Comparative analysis of physical therapy for knee osteoarthritis

The physical therapy methods elucidated in this article, as well as the combination of various physical therapy approaches, have significant value for KOA treatment. There are still unexplored combinations of physical therapy methods. Further research is needed in the future to refine diverse combination approaches for application in KOA. Table 7 provides a comparison of the effects of treating KOA using various physical modalities.

**Table 7:** Comparison of the effects of various physical therapy methods for KOA treatment.

Treatment method	Research design	Treatment efficacy rate	Statistical significance
Acupuncture	Dividing 328 knee osteoarthritis patients into observation and control groups	90.9%	*P* < .05
Moxibustion	Meta-analysis with 746 participants divided into observation and control groups	95%	*P* < .0001
Ultrasonic	46 KOA participants equally divided into 2 groups of 23 each for control analysis	The experimental group showed significant pain relief	*P* < .01
Shortwave	Database meta-analysis	95%	*P* < .05
PEMF	Dividing 24 rabbits into normal, model, and PEMF groups	PEMF can effectively inhibit chondrocyte apoptosis and suppress cartilage degeneration	*P* < .01
Low-intensity laser	Randomized double-blind control for 33 bilateral KOA patients	92%	*P* < .05
Electrotherapy	Dividing 60 KOA patients into acupuncture and electrotherapy groups	91.35%	*P* = .025

KOA = knee osteoarthritis, PEMF = pulsed electromagnetic field.

The *P*-values in the 4th column of Table 7 have statistical significance. When the *P*-value is <.05, the result is considered significant. When the *P*-value is >.05, the result is deemed to lack statistical significance. According to the above comparison, all *P*-values are <.05, indicating that all comparison results have statistical significance and are of reference value. Based on the table’s analysis, moxibustion and electrotherapy are 2 physical therapy methods worth recommending. Specifically, the moxibustion treatment had a high sample size of 746 individuals, with a *P*-value <.0001, signifying a very high statistical significance. The final effective treatment rate reached 95%.

In contrast, theoretically, the shortwave treatment method achieved an effective treatment rate of 95% through meta-analysis in experimental methods, also demonstrating statistical significance. In contrast, there are relatively few literature examples for the experimental cases of PEMF treatment for KOA. Most use animals as experimental subjects, hence lacking clinical validation. For acupuncture treatment, although 328 subjects were tested, since the acupuncture treatment for these 328 subjects was conducted by multiple expert doctors, each with potential variability in technique, the final effective rate for acupuncture treatment for KOA in this table is merely a reference value.

While ultrasound and low-intensity laser treatments don’t have specific effective rates for treating KOA, in their experiments, they were found to effectively alleviate the pain of KOA, indicating their potential as treatment methods for KOA.

Table 8 provides estimated prices based on a survey in the Huzhou region of Zhejiang Province, China. The actual prices should be inquired based on the local policies of the respective regions. Analyzing the data from Table 7, it is evident that among various physical therapy methods, moxibustion and electrotherapy have significant therapeutic value due to their effective treatment rate, convenience, operational difficulty, and treatment costs. Moxibustion is a treatment method involving the fumigation and hovering over acupoints after burning mugwort wool. Those interested can purchase relevant equipment for at-home treatment, offering extreme convenience and relatively low operational difficulty. Compared to acupuncture, ultrasound, shortwave, PEMF, and low-intensity lasers, moxibustion is easier to operate.

**Table 8:** Cost-effectiveness and feasibility of various physical therapies.

Treatment method	Treatment cost	Convenience	Operational difficulty
Acupuncture	50	Highly convenient	Relatively high
Moxibustion	100	Highly convenient	Normal
Ultrasonic	3000	Low convenience	Extremely high
Shortwave	200	Low convenience	Extremely high
PEMF	1000	Low convenience	Extremely high
Low-intensity laser	2000–10,000	Low convenience	Extremely high
Electrotherapy	50	Moderately convenient	Simple

PEMF = pulsed electromagnetic field.

The treatment costs in this table are denominated in Chinese YUAN, and are estimated prices obtained from research in the inland areas of Zhejiang Province, China. The actual prices should be consulted based on the policies of each region.

Electrotherapy is also relatively straightforward, involving treatment via local patches. Hence, precision in targeting acupoints, as required in acupuncture, isn’t essential. It is sufficient to attach the patch approximately over the acupoint, making it convenient and simple. Additionally, the cost of electrotherapy devices is relatively low. Users can undergo electrotherapy at home, with the process being easy and extremely convenient. However, it’s preferable to have someone supervise during treatment. Compared to other treatments, acupuncture is cost-effective and convenient but varies depending on the practitioner. At the same time, acupuncture requires a high level of expertise in traditional Chinese medicine. Advanced technologies involved in ultrasound, shortwave, PEMF, and low-intensity lasers make their treatment costs relatively high, making them less user-friendly.

For individuals, moxibustion and electrotherapy are the primary treatment choices. However, other methods still have their medical value and research potential.

## 4. Conclusion and discussion

KOA is a common degenerative joint disease, predominantly occurring in the elderly population. It severely impacts the quality of life for patients and brings significant burden to families and society. Physical therapy plays an essential role in symptom relief and rehabilitation for KOA patients, enhancing clinical therapeutic outcomes. Moreover, physical therapy can be administered at home, reducing financial pressure. The currently recognized physical treatments with a positive effect on KOA include acupuncture, moxibustion, ultrasound, shortwave, PEMF, low-intensity lasers, and electrotherapy. Analysis and discussion among various physical treatments revealed that moxibustion and electrotherapy are the primary universally applicable treatments, followed by acupuncture. However, other physical treatments need further exploration and research.

Future studies could focus on 3 aspects: For treatments with unclear efficacy and insufficient data, well-designed high-quality randomized controlled trials are required to verify their reliability and therapeutic effect. For novel physical therapies, more clinical data is needed to confirm their authenticity and efficacy. Future studies should determine the optimal treatment prescriptions and parameters for various KOA physical therapies, providing scientific evidence and clinical guidance for patients. The combined physical therapies described in this article have enhanced the therapeutic effect for KOA treatment. Future research can build on this foundation, exploring the best combination models and related parameters and data.

## Author contributions

**Conceptualization:** Zefeng Wang.

**Formal analysis:** Cong Yan.

**Funding acquisition:** Zefeng Wang.

**Investigation:** Xinxin Ni, Minghai Lin, Xinyue Wu, Guangdong Xie, Huajun Wang, Zhengting Cai, Xianwei Lin, Gege Zhang, Dan Wang, Shijia Hu.

**Methodology:** Xinxin Ni, Lianxin Hu, Xun Zhang, Laurent Peyrodie, Xinyue Wu, Guangdong Xie, Zhengting Cai.

**Project administration:** Zefeng Wang, Xun Zhang.

**Resources:** Zefeng Wang, Xun Zhang, Cong Yan.

**Supervision:** Lianxin Hu, Zefeng Wang, Cong Yan, Xun Zhang.

**Validation:** Xinxin Ni, Lianxin Hu, Zefeng Wang, Cong Yan, Laurent Peyrodie, Xun Zhang.

**Writing – original draft:** Xinxin Ni.

**Writing – review & editing:** Xinxin Ni.

## References

[1] Bo L. The impact of somatosensory interactive rehabilitation training on the quality of life of patients with adhesive capsulitis of the shoulder. J Chronic Dis. 2022;7:996–8.

[2] Wei L Zhu M Peng T Xiong W Hou X. Different acupuncture therapies combined with rehabilitation in the treatment of scapulohumeral periarthritis: a protocol for systematic review and network meta-analysis. Medicine (Baltimore). 2020;99:e23085. 33371060 10.1097/MD.0000000000023085 PMC7748163

[3] Huaixing Q Gaojiong Z Fengjing J. Therapeutic effect observation of comprehensive physical therapy for periarthritis of the shoulder. Chin J Phys Med Rehab. 2009;8:559–61.

[4] Xiran F Duoduo L Shuangshuang W. Qualitative study on the influencing factors of treating musculoskeletal pain with tuina technique. Chin General Pract. 2023;26:219–24.

[5] Wallace IJ Worthington S Felson DT. Knee osteoarthritis has doubled in prevalence since the mid-20th century. Proc Natl Acad Sci USA. 2017;114:9332–6. 28808025 10.1073/pnas.1703856114 PMC5584421

[6] Huan W He S Yaonan Z. Survey on the condition of primary osteoarthritis of the knee in different compartments among the Chinese Population Aged 40 and Above. Chin J Bone Joint Surg. 2019;12:528–32.

[7] Changwei J Yaosheng Y. Epidemiological survey of knee osteoarthritis in Jining Region. China Health Standard Manag. 2016;7:9–11.

[8] Kumar H Pal CP Sharma YK Kumar S Uppal A. Epidemiology of knee osteoarthritis using Kellgren and Lawrence scale in Indian population. J Clin Orthop Trauma. 2020;11(Suppl 1):S125–9. 31992932 10.1016/j.jcot.2019.05.019 PMC6977151

[9] Bin W Dan X Shengjie D. Systematic Review on the Epidemiology and Disease Burden of Knee Osteoarthritis in China. Chinese Journal of Evidence-Based Medicine. 2018;18:134–42.

[10] Peng L Meihua G Lixia G. Epidemiological study on osteoarthritis in middle-aged and elderly people. Jilin Med. 2012;33:2576.

[11] Tianchen H Remila A. Progress in clinical physical therapy for knee osteoarthritis. Sichuan Med. 2023;44:888–91.

[12] Zixin Z Qiangling Y Wenjun Y. The application of physical therapy in community rehabilitation for knee osteoarthritis. Chin Community Physicians. 2023;39:130–2.

[13] Sun N Wang LQ Shao JK. An expert consensus to standardize acupuncture treatment for knee osteoarthritis. Acupunct Med. 2020;38:327–34. 32309995 10.1177/0964528419900789

[14] Ben-Arie E Pei-Yu K Yu-Chen L. The effectiveness of acupuncture in the treatment of frozen shoulder: a systematic review and meta-analysis. Evid Based Complement Alternat Med. 2020;2020:9790470. 33062030 10.1155/2020/9790470 PMC7532995

[15] Jinghui Z Yaochi W Yanyan X. The application effect and mechanism of acupuncture in treating knee osteoarthritis. Chin J Tissue Eng Res. 2013;17:5255–60.

[16] Qianru Z Wenbin F. Observational study on the therapeutic effect of combined acupuncture treatment for knee osteoarthritis. Chin Acupunct. 2010;30:375–8.

[17] Shilai Z Yuying H Shumei H. Clinical observations on the efficacy of acupuncture treatment for knee osteoarthritis in 28 Cases. Med Theory Pract. 2016;29:3084–5.

[18] Shuang W Jinyun X Yan W. Effects of moxibustion on the pathological changes of cartilage and behavioral changes in a rabbit model of knee osteoarthritis. Guide Tradit Chin Med Pharm. 2021;27:24–7.

[19] Junchen F Huimin Z Miao Z. Cumulative meta-analysis and trial sequential analysis of moxibustion treatment for knee osteoarthritis. J Nurs Sci. 2018;25:35–43.

[20] Ning L Bin W Yongling Z. Observational study on the efficacy of moxibustion combined with exercise therapy for knee osteoarthritis. Chin Acupunct. 2002;11:9–11.

[21] Xuepu W. Treatment of knee osteoarthritis in 48 cases using warm needle scraping. J North China Coal Med College. 2005;7:199–199.

[22] Yuan T Xiong J Wang X. The effectiveness and safety of moxibustion for treating knee osteoarthritis: a PRISMA compliant systematic review and meta-analysis of randomized controlled trials. Pain Res Manag. 2019;2019:2653792. 31949547 10.1155/2019/2653792 PMC6935827

[23] Dai M Fang X Chen H Wang Y-hong Wu Y-wen. Clinical study on mild moxibustion for knee osteoarthritis. J Acupunct Tuina Sci. 2018;17:62–6.

[24] Guangji Z Qingwei W Junji L. Treatment of rabbit abdominal skin scars with ultrasound of different intensities. Chin J Tissue Eng Res. 2023;27:3640–5.

[25] Xianwen L Mingxing L. Low-intensity pulsed ultrasound can alleviate knee osteoarthritis pain and repair joint cartilage damage. Chin J Tissue Eng Res. 2019;23:348–53.

[26] Yuebin L Tihao X Yugang H. The effect of traditional Chinese medicine acupuncture combined with ultrasound treatment on pain level and joint function recovery in patients with knee osteoarthritis. Famous Doctor. 2020;99:89–90.

[27] Haitao Z Qiulian W. Clinical observation on the treatment of knee osteoarthritis with warm acupuncture combined with ultrasound. Chin Med J Guide. 2014;11:84–6.

[28] Özgönenel L Aytekin E Durmuşoǧlu G. A double-blind trial of clinical effects of therapeutic ultrasound in knee osteoarthritis. Ultrasound Med Biol. 2009;35:44–9. 18829151 10.1016/j.ultrasmedbio.2008.07.009

[29] Yang P Li D Zhang S. Efficacy of ultrasound in the treatment of osteoarthritis of the knee. Orthopaedic Surg. 2011;3:181–7. 10.1111/j.1757-7861.2011.00144.x PMC6583622 22009649

[30] Yeğin T Altan L Aksoy MK. The effect of therapeutic ultrasound on pain and physical function in patients with knee osteoarthritis. Ultrasound Med Biol. 2017;43:187–94. 27727020 10.1016/j.ultrasmedbio.2016.08.035

[31] Draper DO Klyve D Ortiz R Best TM. Effect of low-intensity long-duration ultrasound on the symptomatic relief of knee osteoarthritis: a randomized, placebo-controlled double-blind study. J Orthop Surg Res. 2018;13:1–9. 30326947 10.1186/s13018-018-0965-0 PMC6192104

[32] Yu C. Clinical efficacy observation of ultrashort wave treatment for knee osteoarthritis. Chin J Pract Neurol Dis. 2009;12:75.

[33] Xiaohui H. Observation of the therapeutic effect of intra-articular injection combined with shortwave therapy for knee osteoarthritis. Massage Rehab Med. 2017;8:61–2.

[34] Hongyan J Jinxiu D. Improvement of knee osteoarthritis in 30 cases by acupuncture combined with ultrashort wave. Chin Modern Dist Educ Trad Chin Med. 2014;12:14–6.

[35] Huipeng Y. efficacy observation of ultrashort wave combined with tuina technique in treating knee osteoarthritis. J Changchun Univ Tradit Chin Med. 2011;27:453–4.

[36] Rutjes AWS Nueesch E Sterchi R. Therapeutic ultrasound for osteoarthritis of the knee or hip. Cochrane Database Syst Rev. 2010;10:1002. 10.1002/14651858.CD003132.pub2 20091539

[37] Shields N Gormley J O’Hare N. Short-wave diathermy: current clinical and safety practices. Physiother Res Int. 2002;7:191–202. 12528575 10.1002/pri.259

[38] Bao Z Fan M Ma L Duan Q Jiang W. The effects of pulsed electromagnetic fields combined with a static magnetic intramedullary implant on the repair of bone defects: a preliminary study. Electromagn Biol Med. 2019;38:210–7. 31155966 10.1080/15368378.2019.1625785

[39] Fan Y. Application of pulsed electromagnetic field therapy in the treatment of bone diseases. China Rehab Theory Pract. 1999;3:37–9.

[40] Chen C Ying M Yan J. Meta-analysis on the efficacy of low-intensity and high-intensity laser combined with rehabilitation exercises for knee osteoarthritis. China Modern Drug Appl. 2023;17:12–8.

[41] Li S Nan S Wei L. Observational study on the efficacy of acupuncture combined with laser treatment in 89 cases of knee osteoarthritis. J China Endemic Dis Prev. 2012;27:474–8.

[42] Xiangling L Xiaoyun C Nalai J. Clinical comparative study on the treatment of knee osteoarthritis with acupuncture and helium-neon laser. Shanghai J Acupunct. 2012;31:829–30.

[43] Alfredo PP Bjordal JM Lopes-Martins RB. Efficacy of prolonged application of low-level laser therapy combined with exercise in knee osteoarthritis: a randomized controlled double-blind study. Clin Rehabil. 2022;36:1281–91. 35918813 10.1177/02692155221111922

[44] Xiang D Yi Z Zhenhan D. Meta-analysis of transcutaneous electrical nerve stimulation for the treatment of pain in knee osteoarthritis. Chin J Tissue Eng Res. 2015;19:1798–804.

[45] Xia J. The application effect of low-frequency physical therapy and its influence on limb mobility in patients with knee osteoarthritis. Chin Sci Technol J Database (Citation Edition) Med Health. 2023;6:0035–7.

[46] Chunyan L Xuelong T Xuehong Y. Design of medium-low frequency electrotherapy and pain assessment system. J Biomed Eng. 2014;31:558–62. 25219234

[47] Zeng C Li H Yang T. Electrical stimulation for pain relief in Knee osteoarthritis; systematic review and network meta-analysis. Osteoarthritis Cartilage. 2015;23:189–202. 25497083 10.1016/j.joca.2014.11.014

[48] Xiaojun J Neng C Jinzhi L. Clinical study on the formulation and optimization of comprehensive intervention scheme for elderly knee osteoarthritis with integration of traditional Chinese and Western medicine. Adv Modern Biomed. 2022;22:1375–9.

[49] Ming L Xiang L Xiaoping C. Observation of the analgesic effect of low to medium frequency therapy device on knee osteoarthritis. Chin Sci Technol J Database (Full-text Edition) Med Health. 2022;5:0009–12.

[50] Fields HL Basbaum AI. Central nervous system mechanisms of pain modulation, in Wall PD Melzack R (eds): Textbook of Pain, chap 12. New York, NY: Wall and Melzack’s textbook of pain; 1999:243–57.

[51] Hui OY Xiubao S Yuping W. Efficacy of comprehensive rehabilitation therapy on bone marrow edema in mild to moderate knee osteoarthritis. J Jinan Univ (Natural Sci Med Edition). 2015;36:88–91.

[52] Yanmei G Lining Z. Observation on the efficacy of high-frequency electrical stimulation combined with exercise training in the treatment of knee osteoarthritis. Chin J Rehab Theory Pract. 2009;15:168–9.

[53] Zhijiao F Yubao M Shuyan Q. The effect of electrotherapy of different intensities on the functional recovery of patients with knee osteoarthritis. Chin J Geriatric Health Care Med. 2022;20:66–9.

[54] Junshuang W. Observation of the analgesic effect of low and medium frequency electrotherapy on knee osteoarthritis. Clin Med Literature Electronic J. 2019;6:49.

[55] Shim JW Jung JY Kim SS. Effects of electroacupuncture for knee osteoarthritis: a systematic review and meta-analysis. Evid Based Complement Alternat Med. 2016;2016:3485875. 27818699 10.1155/2016/3485875 PMC5081971

[56] Erhong Y Qiuhong M. Observation of the therapeutic effect of warm acupuncture combined with shortwave therapy in 60 cases of knee osteoarthritis. Inner Mongolia J Tradit Chin Med. 2014;33:36–7.

[57] Yanling Z Xiaoxiu X Chun L. The effect of warm acupuncture combined with shortwave therapy on TNF-α and IL-6 levels in patients with knee osteoarthritis. Ningxia Med J. 2018;5.

[58] Al Rashoud AS Abboud RJ Wang W. Effcacy oflow – level laser therapy applied at acupuncture points in kneeosteoarthritis: a randomised double – blind comparative trial. Physiotherapy. 2014;100:242e8. 24418801 10.1016/j.physio.2013.09.007

[59] Lin L Ke C Xueyong S. A clinical research review on the treatment of knee osteoarthritis using low-intensity laser acupuncture in the recent 5 years. Chin J Tradit Chin Med. 2020;38:104–8.

[60] Plaster R Vieira WB Alencar FAD Nakano EY Liebano RE. Immediate effects of electroacupuncture and manual acupuncture on pain, mobility and muscle strength in patients with knee osteoarthritis: a randomised controlled trial. Acupunct Med. 2014;32:236–41. 24566612 10.1136/acupmed-2013-010489

